# Supplementary material for: An optogenetic toolkit for light-inducible antibiotic resistance
Source: Nat Commun. 2023 Feb 23;14:1034. doi: 10.1038/s41467-023-36670-2 (PMC9950086; doi:10.1038/s41467-023-36670-2)
Supplement: Supplementary file 1 — Supplementary Information [file 41467_2023_36670_MOESM1_ESM.pdf]

SUPPLEMENTARY INFORMATION

An Optogenetic Toolkit for Light-Inducible Antibiotic Resistance

Michael B. Sheets,[1,2] Nathan Tague,[1,2] Mary J. Dunlop[1,2,*]

[1] Department of Biomedical Engineering, Boston University, Boston, Massachusetts 02215, United States

[2] Biological Design Center, Boston University, Boston, Massachusetts 02215, United States

* corresponding author: mjdunlop@bu.edu

This PDF file includes:

Supplementary Figure 1: Optogenetic activation of OptoCre-*bla* and OptoCre-*tetA* on plasmid.
Supplementary Figure 2: Tuning OptoCre-*cat* by changing light intensity and duration.
Supplementary Figure 3: Characterization of synthetic terminators.
Supplementary Figure 4: Growth recovery of cells from time-course microscopy.
Supplementary Figure 5: Microscopy of resistance activation using a digital micromirror device.
Supplementary Figure 6: Growth time-course for octanoic acid production strains.

Supplementary Table 1: Primers used for plasmid insertion of antibiotic resistance constructs.

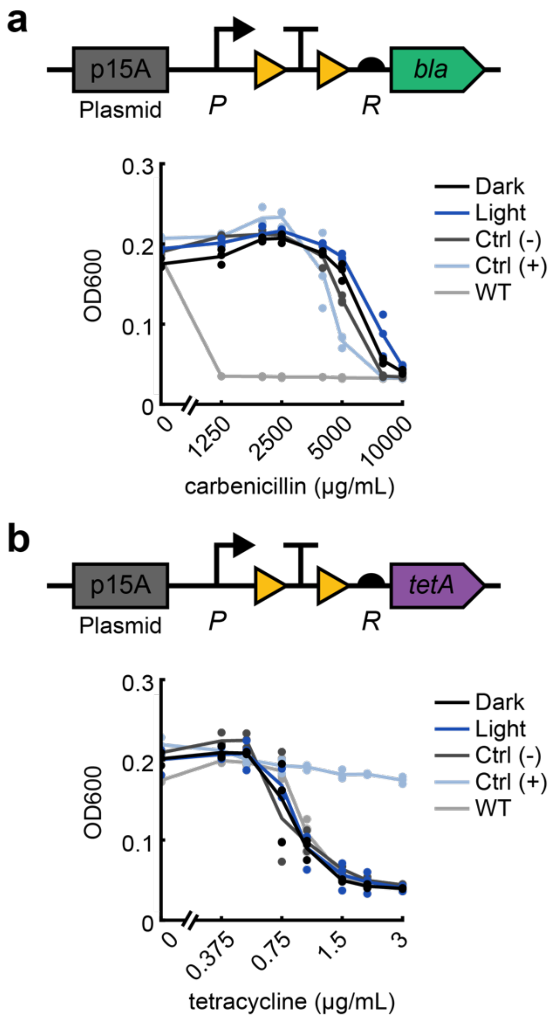

Supplementary Figure 1. Optogenetic activation of **(a)** OptoCre-*bla* and **(b)** OptoCre-*tetA* on the p15A plasmid origin using promoter *P* and RBS *R*. MIC is quantified by OD600 after 18 hours (n = 3 biological replicates).

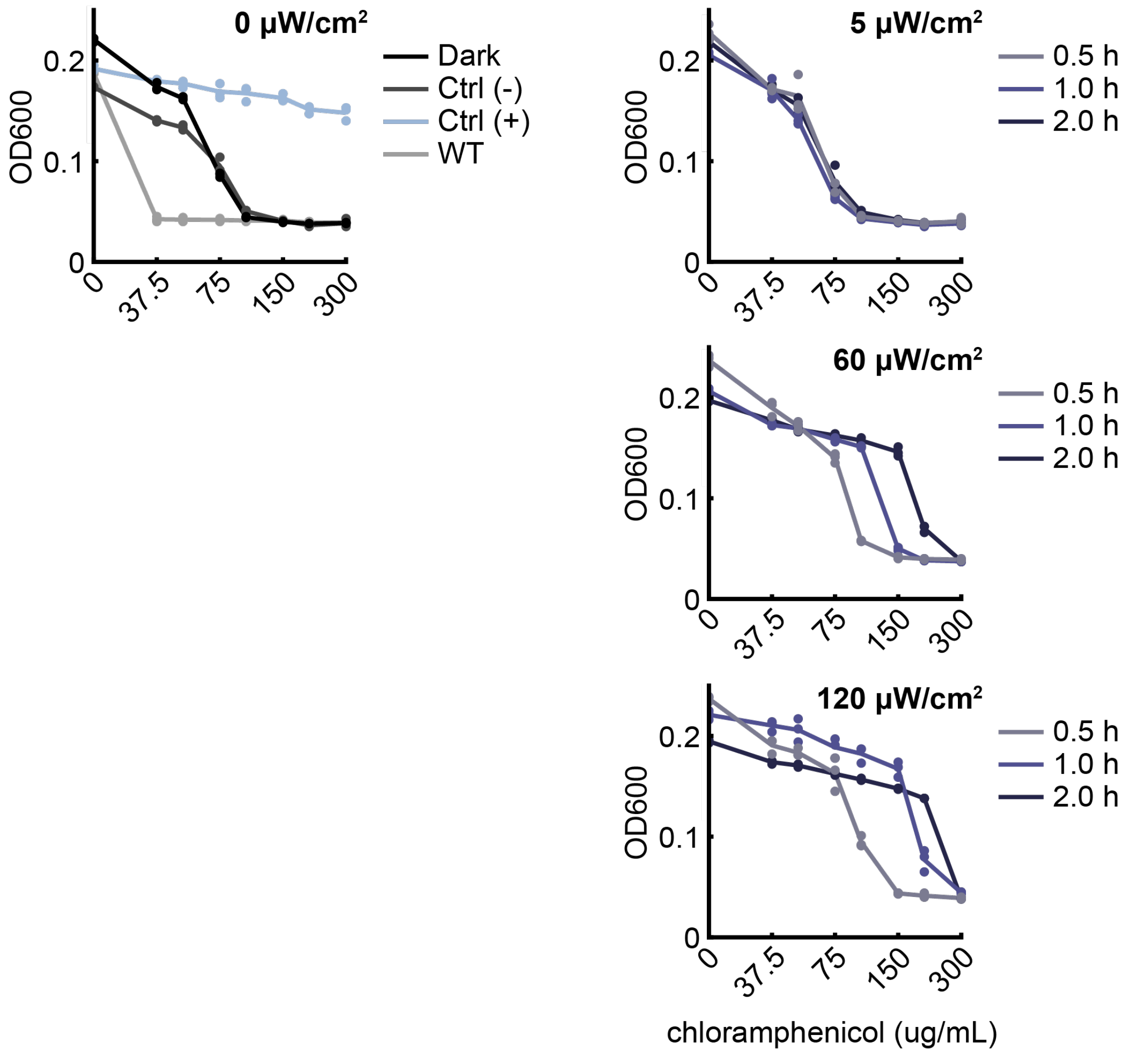

Supplementary Figure 2. Tuning chloramphenicol resistance by changing light intensity and duration with p15A plasmid-based OptoCre-*cat* using *cat*$_{T172A}$ with promoter P and RBS R. MIC is quantified by OD600 after 18 hours (n = 3 biological replicates).

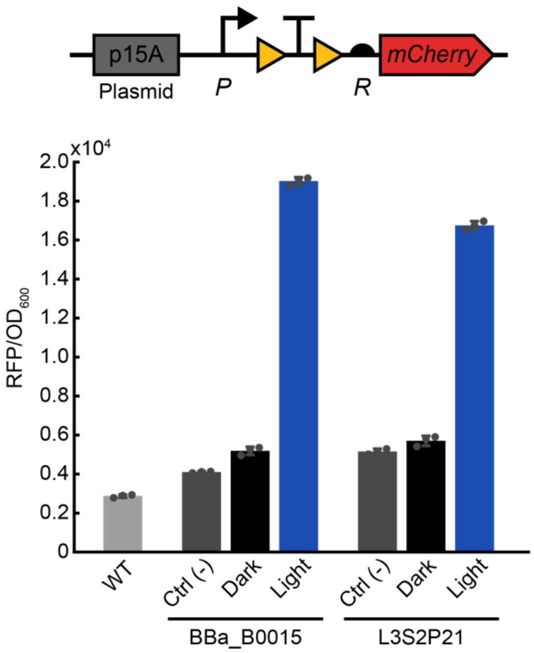

Supplementary Figure 3. Characterization of synthetic terminators BBa_B0015 and L3S2P21 using mCherry fluorescence (n = 3 biological replicates). Terminator BBa_B0015 is used in all OptoCre antibiotic activation cassettes.

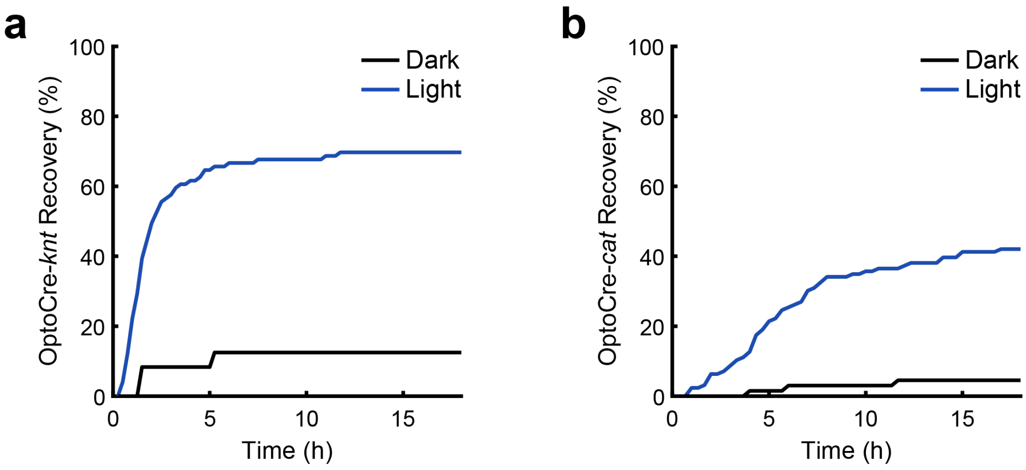

Supplementary Figure 4. Growth recovery of cells from time-course microscopy of **(a)** chromosomal OptoCre-*knt* with promoter P* and RBS R on agarose pads containing 400 µg/mL kanamycin, and **(b)** p15A plasmid-based OptoCre-*cat* using cat_{T172A} with promoter P and RBS R on agarose pads containing 60 µg/mL chloramphenicol. Recovery is measured as percent cells present in the first frame that have divided at or before a given time point. Measurements are cumulative across three imaging positions for each condition.

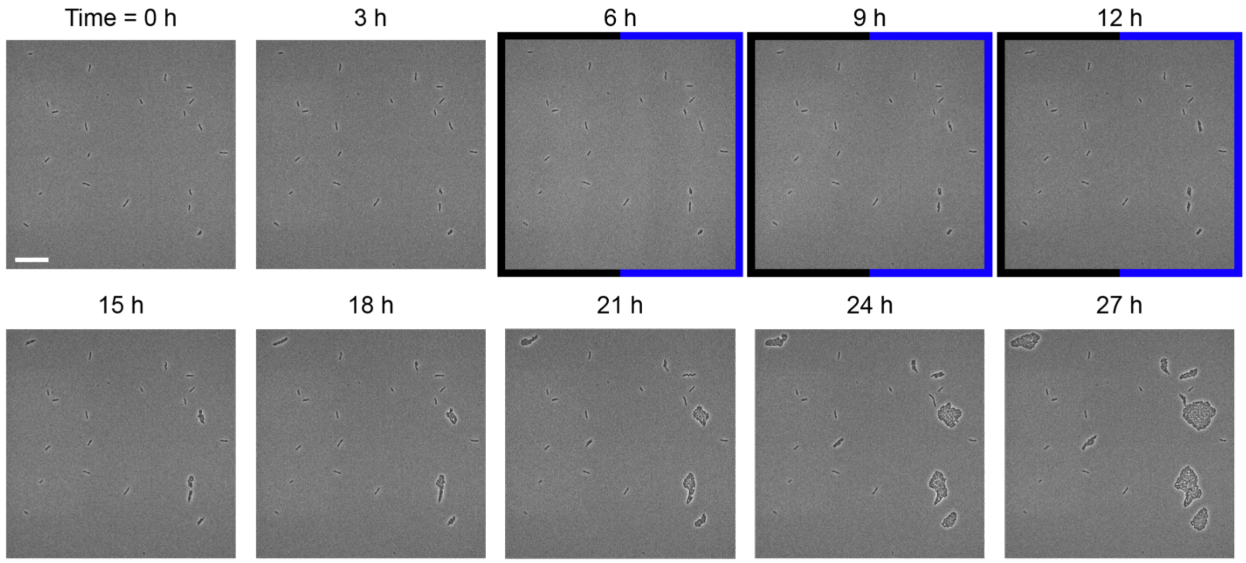

Supplementary Figure 5. Single-cell time lapse microscopy of resistance induced by illuminating half of the field of view with a digital micromirror device (DMD). Activation of p15A plasmid-based OptoCre-*cat* using cat_{T172A} with promoter P and RBS R on agarose pads containing 60 µg/mL chloramphenicol (scale bar = 10 µm). DMD light activation was carried out on the right half of the frame from hours 6 to 12 of the experiment. This experiment was repeated three times on separate days, with similar results for all experiments.

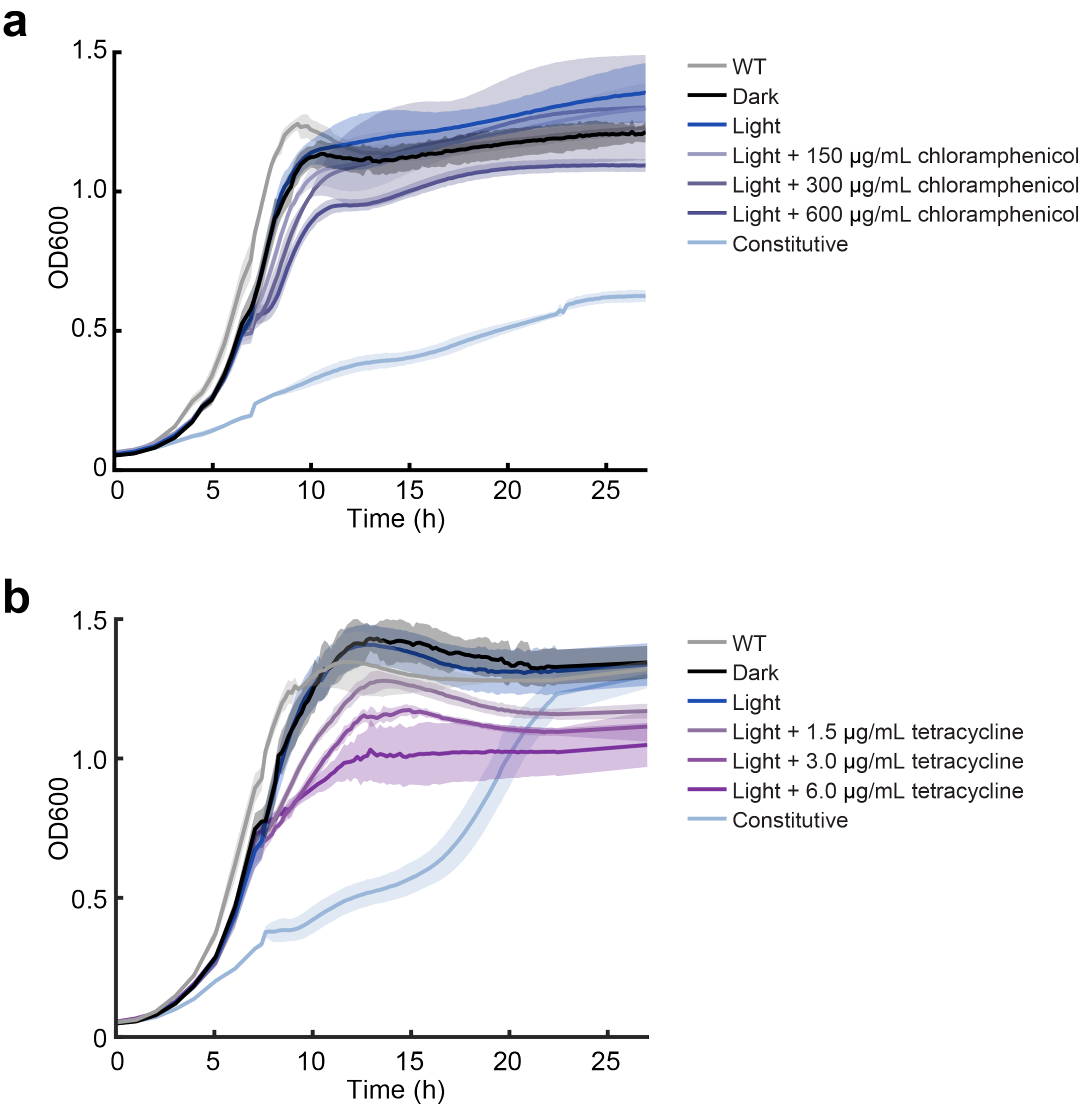

Supplementary Figure 6. Growth time-course for octanoic acid production strains. **(a)** Activation of p15A plasmid-based OptoCre-*cat* with promoter P and RBS R and cat_{T172A}, and RBS R' expressing *Cp*FatB1. Light induction was from 4.5-6.5 hours, with chloramphenicol added at 6.5 hours for the indicated strains. **(b)** Activation of p15A plasmid-based OptoCre-*tetA* with promoter P_{tet} and RBS R_{tet} expressing *tetA*, and RBS R' expressing *Cp*FatB1. Light induction was from 4-6 hours, with tetracycline added at 6 hours for indicated strains. Shaded error bars show standard deviation around the mean (n = 3 biological replicates).

Supplementary Table 1. Primers used for plasmid insertion of antibiotic resistance constructs using either Gibson or Golden Gate (GG) method. Binding regions in uppercase.

Element	Assembly Method	Forward Primer	Reverse Primer
bla	Gibson	aagaaggagatatacatATGAGTATTCAACATTTCCG	tgccctggagatccctattaCCAATGCTTAATCAGTGAG
knt	Gibson	tttaagaaggagatatacatATGATTGAACAAGATGGATTGC	atgcctggagatccctattaTCAGAAGAACTCGTCAAGAAG
cat	Gibson	tttaagaaggagatatacatATGGAGAAAAAAATCACTGGAT	atgcctggagatccctattaTTACGCCCCGCCC
tetA (loxP-TT-loxP insertion)	GG	gtgactcgtctcgGCACGGCGAAATAACTTC	gtgactcgtctcgcgCTTCTTAAAATAACTTCGTATAATG
*Cp*FatB1	Gibson	gggcgtaataatagggatctTTTCAGAATTCAAAAGATCTTTT	atgcctggagatccctattaATGCCTGGAGATCCTTACTC
P*	GG	tacgctggtctccttGTCTTAAAGTCTAACCTATAGGATTCTTAC	tacgctggtctccgtCCCTCTCGATGGCTGTAAGA
P**	GG	tacgctggtctccttGTAATAAAGTCTAACCTATAGGATTTTTAC	tacgctggtctccgtCCCTCTCGATGGCTGTAAAA
R*	GG	gcatgaggtctcctaGCATACATTATACGAAGTTATATCACTCTACGG	cgatcaggtctcgaaTCATGTTTGCAGCTGGCCG
R’	GG	agtggaggtctcctcTCTTTTGTTTAATTACTAAGCGGGAGGTTAT	agtggaggtctcctcATAACCTCCCGCTTAGTAATTAAACAAA
L3S2P21	GG	gatgtccgtctccTCCTCGGTACCAAATTCCAGAAA	gatgtccgtctccGCAGGACCAAAACGAAAAAAGGC
Plasmid resistance marker swaps	Gibson	GATCTATCAACAGGAGTCCAAGC	GCGCAACGCAATTAATGTAAGT
FRT cassette addition (cassette)	Gibson	gttttgcgccattcgatggtGCAGCATTACACGTCTTGAG	acatcaccgatggggaagatcctgtcaaacatgagaattaattccg
FRT cassette addition (vector)	Gibson	ttaattctcatgtttgacagGATCTTCCCCATCGGTGATGTC	ctcaagacgtgtaatgctgCACCATCGAATGGCGCAAAAC
