# Supplementary material for: An optogenetic toolkit for light-inducible antibiotic resistance
Source: Nat Commun. 2023 Feb 23;14:1034. doi: 10.1038/s41467-023-36670-2 (PMC9950086; doi:10.1038/s41467-023-36670-2)
Supplement: Supplementary file 2 — Description of Additional Supplementary Files [file 41467_2023_36670_MOESM2_ESM.pdf]

Title: Supplementary Movie 1.
Description: Time-lapse microscopy of light-induced chromosomal OptoCre-*knt* with promoter P* and RBS R on agarose pads containing 400 µg/mL kanamycin (scale bar = 10 µm). Images show a representative position of the OptoCre-*knt* activation strain without (left) or with (right) blue light. Light is provided by an LED light ring above the microscope stage, and exposure begins immediately after cells are added to antibiotic-containing pads.

Title: Supplementary Movie 2.
Description: Time-lapse microscopy of light-induced p15A plasmid-based OptoCre-*cat* using cat_{T172A} with promoter P and RBS R on agarose pads containing 60 µg/mL chloramphenicol (scale bar = 10 µm). Images show a representative position of the OptoCre-*cat* activation strain without (left) or with (right) blue light. Light is provided by an LED light ring above the microscope stage, and exposure begins immediately after cells are added to antibiotic-containing pads.
